# The Effect of Birthing Ball Exercises on Labor Pain and Labor Outcome Among Primigraviade Parturient Mothers at a Tertiary Care Hospital

**DOI:** 10.7759/cureus.36088

**Published:** 2023-03-13

**Authors:** Sujata Jha, Himanshu Vyas, Mamta Nebhinani, Pratibha Singh, Deviga T

**Affiliations:** 1 Nursing, All India Institute of Medical Sciences, Bhopal, IND; 2 Nursing, All India Institute of Medical Sciences, Jodhpur, IND; 3 Obstetrics & Gynecology, All India Institute of Medical Sciences, Jodhpur, IND

**Keywords:** birthing ball exercise, primigravidae, neonatal outcome, maternal outcome, labour pain

## Abstract

Introduction: Every woman has the right to respectful and empathetic care during childbirth that addresses her needs for pain management, and allows her the liberty to make it a memorable experience. This study aimed to assess the effect of birthing ball exercises on labor pain and labor outcome among primigravidae parturients at a tertiary care hospital.

Method: A quasi-experimental design was used. A total of 60 primigravidae with 30 each in the control and experiment groups were selected by consecutive sampling. Primigravidae in the experiment group underwent two sessions of 20 minutes of birthing ball exercises at a subsequent gap of one hour during their active phase of labor (>4 cm cervical dilation). Primigravidae in the control group received routine standard care that included continuous observation and monitoring of vital signs and progress of labor. The visual analog scale (VAS) score was assessed in the transition phase (cervical dilation 8 cm to 10cm) and labor outcomes were assessed after delivery in both groups.

Result: The experiment group had significantly better labor outcomes in terms of labor pain, cervical dilatation, and duration of labor compared to the primigravidae in the control group (p<0.05). In addition, the majority of mothers in the experiment group (86.7 %) underwent vaginal delivery with episiotomy compared to the control group (53.3%). Findings also revealed a statistically significant difference in the newborns of both groups regarding appearance, pulse, grimace, activity, and respiration* *(APGAR) score, crying immediately after birth, and admission to the neonatal intensive care unit (NICU) at p<0.05.

Conclusion: There are a variety of discomforts that a woman experiences during labor. Reducing these discomforts is an important part of good nursing care. Non-pharmacologic methods like birthing ball exercises help decrease these discomforts by reducing labor pain and improving maternal and neonatal outcomes.

## Introduction

Pregnancy and childbirth are important events in a woman’s life. Both pregnancy and childbirth are physiological events and the body of the female is prepared to conceive carry and give birth to a baby. Labor pain though physiological can be very challenging for women to bear. Every woman expects respectful and empathetic care during childbirth that addresses pain management and allows her the liberty to make it a memorable experience [1].

The complete process of labor is divided into four stages. The first stage starts with the onset of true labor pains and is also known as the cervical stage of labor. This is the longest stage of labor and may extend up to 12 hours in primigravidae. The intensity and frequency of labor pains increase with the progression of labor and at times the pain becomes unbearable to the women.

Addressing labor pains is a very important intervention and the birthing ball exercises can prove to be an important tool for the same. A birthing ball is an air-filled elastic ball that provides a broad base to the mother for sitting and enables an upright position [2]. There is an emphasis on non-pharmacological aspects to reduce the pain of laboring women. The use of a birthing ball can play a vital role as a non-pharmacological method of pain relief, as it can also aid in enhancing the positive birth experience by achieving good labor progress and better labor outcomes. The birthing ball offers a counterforce on the perineum and escalates the cervical dilatation and also broadens the pelvic outlet [3].

Evidence suggests that light massage and counter pressure provide relief to many women during the first stage of labor [4]. Birthing ball exercises help bring the fetal longitudinal axis to fall in line with the birth axis which eventually shortens the duration of the first stage of labor and favors vaginal delivery [5].

The birthing ball facilitates upright positioning during labor that promotes the descent of the fetus aided by gravity as well as increased pelvic dimensions [6]. Women engaged in birthing ball exercises also feel empowered psychologically as they take an active role in their own care during labor which helps them improve control over body posture and aids in dealing with labor pain [7]. Therefore, this study aims to explore the effectiveness of birthing ball exercises on labor pain and labor outcome among primigravidae mothers.

## Materials and methods

Study design

A quasi-experimental design was used to study a total of 60 primigravidae with 30 in each group i.e., the control group and the experiment group. The participants were selected by consecutive sampling.

The inclusion criteria for sample selection were: primigravidae parturient mothers (age 18 to 35years) with spontaneous labor, in the active phase of labor (>4 cm cervical dilatation), gestation age more than 37 weeks, and singleton fetus with cephalic presentation. Primigravidae mothers with high-risk pregnancies, conceived after treatment of infertility, and mothers who are obese, overweight, and short in stature with increased risk of falls or physically challenged were excluded from the study.

Instruments

The tool used for the present study comprised three sections: (I) socio-demographic data collected through interviews via self-structured questionnaire; (II) labor pain assessed using the visual analog scale (VAS) score in which 0=no pain, 1 to 3=mild pain, 4 to 6=moderate pain, 7 to 9=severe pain, and 10=worst pain; (III) labor outcome included maternal outcome (duration of labor, rate of cervical dilatation, rupture of membrane, analgesics given during labor, vital sign during labor, mode of delivery), and neonatal outcome (appearance, pulse, grimace, activity, and respiration (APGAR) score, newborn crying immediately after birth, the neonatal intensive care unit (NICU)) that were measured through partograph and APGAR score, respectively.

Intervention protocol

A birthing ball (65 cm) was provided by the researcher to the primigravidae mother (experimental group) in the active phase of the first stage of labor (i.e., >4 cm cervical dilatation) and primigravidae mothers were encouraged to follow four exercises in two different positions.

The exercises included pelvic rocking forward and backward, ball bouncing and ball hip circle in a sitting position, and hugging the ball in a kneeling position for 20 minutes. There were two, 20-minute exercise sessions with a subsequent gap of one hour during the active phase of labor. Mothers were allowed to change their position in between exercises whenever they want.

After the exercise sessions, the labor pain was assessed using the VAS when cervical dilatation was at 8 cm to 10 cm in both groups. Labor outcome was assessed by using the partograph and APGAR score. The birthing ball exercise sessions were provided in the labor room.

Statistical analysis

Data were cleaned and organized. Descriptive and inferential statistics were used for the analysis of data. A p-value of <0.05 was considered statistically significant.

Ethical approval

Ethical clearance was obtained from the institutional ethics committee of the All India Institute for Medical Sciences, Jodhpur, India (approval no. AIIMS/IEC/2020-2021/3081). The participants gave their consent and were provided with both written and verbal information about the study.

## Results

The results of this study showed that the mean age of the primigravidae experiment group was 24.4±3.68 years whereas, in the control group, it was 24.5±3.63. The mean gestation age (in weeks) of the experiment group was 39.7±1.11 whereas, in the control group, it was 39.4±1.28. No statistically significant difference was observed in the sample characteristics of the primigravidae in the experiment and control groups (Table 1).

**Table 1:** Frequency and percentage distribution of samples on demographic & obstetric variables in the control and experiment groups f: Frequency, df: Degree of freedom, NS: Not significant, SD: Standard deviation

S.No.	Socio-demographic variable	Control group (n=30) f (%)	Experiment group (n=30) f (%)	χ2	df	p-value
1	Age group in years			0.144	2	0.930 (NS)
	18-23	12 (40%)	13 (43.3%)			
	24-29	16 (53.3%)	15 (64%)			
	30-35	2 (6.7%)	2 (6.7%)			
	Means ± SD	24.5±3.63	24.3±3.68			
2	Gestational age in weeks			4.887	2	0.086 (NS)
	37-39 weeks	12 (40%)	7 (23.3%)			
	39.1-41 weeks	14 (46.7%)	19 (63.3%)			
	41.1-42 weeks	4 (13.3%)	4 (13.3%)			
	Mean ± SD	39.4±1.28	39.7±1.11			
3	Education			1.476	3	0.687 (NS)
	No formal education	1 (3.3%)	-			
	Primary	4 (13.3%)	6 (20%)			
	Secondary	7 (23.3%)	6 (20%)			
	Graduation or above	18 (60%)	18 (60%)			
4	Occupation			-	-	-
	Homemaker	29 (96.7%)	26 (86.7%)			
	Private job/self-employed	1 (3.3%)	4(13.3%)			
5	Religion			-	-	-
	Hindu	28 (93.3%)	27 (90%)			
	Muslim	2 (6.7%)	2 (6.7%)			
	Any others	-	1 (3.3%)			
6	Duration of marriage			4.960	2	0.083 (NS)
	<1 year	5 (16.7%)	5 (16.7%)			
	1-3 years	23 (76.7%)	18 (60%)			
	>3 years	2 (6.7%)	7 (23.3%)			
7	Types of family			1.542	2	0.462 (NS)
	Nuclear	2 (6.7%)	3 (10%)			
	Joint	25 (83.3%)	22 (73.3%)			
	Extended	3 (10%)	5 (16.7%)			
8	Monthly income of the family			0.792	2	0.673 (NS)
	<20,000	4 (13.3%)	3 (10%)			
	20,000-30,000	8 (26.7%)	10 (33.3%)			
	>30,000	18 (60%)	17 (56.7%)			

Data presented in Table 2 represents 70% of primigravidae parturient mothers in the control group who reported worst pain, and 30% who reported severe pain. In contrast, in the experiment group, only 16.7% expressed their pain as the worst and 83.3% reported severe pain.

**Table 2:** VAS score of the control and experiment groups (n=60) f: Frequency, VAS: Visual analog scale

S.No.	Level of pain (VAS score)	Control group (n=30) f (%)	Experiment group (n=30) f (%)
1.	Severe pain (7-9)	9 (30%)	25 (83.3%)
2.	Worst pain (10)	21 (70%)	5 (16.7%)

Data presented in Table 3 shows the comparison of labor pain (VAS Score) in the control and experiment groups. The mean and standard deviation (SD) of the VAS score in the control group was 9.4±1.13 as compared to the experiment group where the mean VAS score was 8.36±.97. Unpaired t-test was used to test the hypothesis. A significant difference at p<0.05 was observed in the level of labor pain (VAS score) of the experiment and control groups, and it was concluded that primigravidae parturient mothers who had used the birthing ball during the first stage of labor had reduced intensity of labor pain.

**Table 3:** Mean and standard deviation of pain scores (VAS) in the control and experiment groups df: Degree of freedom, SD: Standard deviation, VAS: Visual analog scale

	Labor pain (VAS score) Mean±SD	df	t-value	p-value
Control group (n=30)	9.4±1.13	58	3.491	0.0010*
Experiment group (n=30)	8.36±.97			

The mean duration of labor in hours among primigravidae in the experiment group was 14.4±3.90 compared to 19.2±3.17 in the control group. The majority of mothers in the experiment group (86.7%) underwent vaginal delivery with episiotomy compared to the control group (53.3%). A significant difference was observed in the duration of labor, rate of cervical dilatation, augmentation/induction of labor, use of analgesics, and mode of delivery among primigravidae in the experiment and control groups at p<0.05 (Table 4).

**Table 4:** Comparison of maternal outcomes in the control group and experiment group (n=60) f: Frequency, df: Degree of freedom, NS: Not significant, Cp: Cerviprime

S.No.	Items	Control group (n=30) f (%)	Experiment group (n=30) f (%)	χ2/Fisher exact	df	p-value
1	Duration of labor (in hours) 8-12 hours	1 (3.3%)	9 (30%)	20.07	3	0.000 (S)
	12-16 hours	3 (10%)	11 (36.7%)			
	16-20 hours	12 (40%)	6 (23.3%)			
	20-24 hours	14 (46.7%)	3 (10%)			
	Means±SD	19.2±3.17	14.4±3.90			
2	Rate of cervical dilatation			27.09	2	0.000 (S)
	<1 cm/hour	23 (76.7%)	4 (13.3%)			
	1 cm/hour	6 (23.3%)	11 (36.7%)			
	>1 cm/hour	1 (3.3%)	15 (50%)			
3	Rupture of membrane			1.195	2	0.550 (NS)
	Spontaneous	11 (36.7%)	13 (43.3%)			
	Artificial	18 (60%)	17 (56.7%)			
	Premature rupture of membrane	1 (3.3%)	-			
4	Augmentation/induction of labor			16.30	2	0.000 (S)
	Not done	-	8(26.7%)			
	Done with oxytocin	17 (56%)	20 (66.7%)			
	Done with Cp gel	13 (44%)	2 (6.7%)			
5	Analgesics during labor			22.05	2	0.000 (S)
	Given	15 (50%)	1 (3.3%)			
	Not given	3 (10%)	17 (56.7%)			
	If any special medication	12 (40%)	12 (40%)			
6	Maternal vitals during labor			2.068	1	0.150 (NS)
	Stable	28 (93.3%)	30 (100%)			
	Not stable	2 (6.7%)	-			
7	Mode of delivery			8.380	1	0.03 (S)
	Vaginal delivery with episiotomy	16 (53.3%)	26 (86.7%)			
	Forceps vaginal delivery	1 (3.3%)	-			
	Vacuum vaginal delivery	1 (3.3%)	-			
	Cesarean section	12 (40%)	4 (13.3%)			

Data presented in Table 5 shows that almost all (96.7%) newborns born to the primigravidae in the experiment group had an APGAR score between 7-10 as compared to 76.7% in the control group. A significant difference was observed in the APGAR score, crying immediately after birth, and requirement of admission to NICU among newborns of primigravidae in the experimental and control groups at p<0.05 (Table 5). No personal variable was found to be significantly associated with labor pain and labor outcome in the control and experimental group at p<0.05.

**Table 5:** Assessment and comparison of neonatal outcome in the control group and experiment group (n=60) f: Frequency; df: Degree of freedom; S: Significant; APGAR: Appearance, pulse, grimace, activity, and respiration; NICU: Neonatal intensive care unit

	Items	Control group (n=30) f (%)	Experimental group (n=30) f (%)	χ2	df	p-value
1.	APGAR score			6.025	2	0.04 (S)
	7-10	23 (76.7%)	29 (96.7%)			
	4-6	5 (16.7%)	-			
	1-3	2 (6.7%)	1 (3.3%)			
2.	Cried immediately after birth			4.138	1	0.04 (S)
	Yes	27 (90%)	29 (96.7%)			
	No	3 (10%)	1 (3.3%)			
3.	Admission to NICU			9.310	1	0.002 (S)
	Yes	4 (13.3%)	1 (3.3%)			
	No	26 (86.7%)	29 (96.7%)			

## Discussion

The current study was conducted to assess the effect of birthing ball exercises on labor pain and labor outcome during the first stage of labor among primigravidae parturient mothers at a tertiary care hospital. This study showed there were no significant differences observed among control and intervention groups regarding general characteristics and obstetric history. This means that both groups were similar in characteristics before the intervention. Results were supported by the quasi-experimental study conducted by Sheishaa et al. to assess the effect of birthing ball exercises during pregnancy on the first stage progress of labor in Egypt [8].

The finding of the present study revealed that there was a statistically significant difference observed between the control and experiment group level of labor pain measured by the VAS score (t=3.491, p<0.05). Similar results were shown in a randomized controlled trial (RCT) conducted by Meei-Ling Gau et al., where the effectiveness of a birth ball exercise program during childbirth was investigated by measuring childbirth self-efficacy and childbirth pain in Taiwan [9].

The current study revealed that the mean and SD of the duration of labor in hours among primigravidae in the experiment group was 13.8±3.55 and in the control group was 19.2±2.76. A significantly shorter duration of labor was observed in the experiment group as compared to the control group. Farrag et al. studied nulliparous women in a maternity unit located at the El-Nabawy El Mohandes Hospital in Egypt and showed similar results [3]. Contrary to the findings of our study, Simin et al. did not find any difference in the duration of the active stage of labor among the two groups [10].

The majority of mothers in the experiment group (86.7%) underwent vaginal delivery with episiotomy compared to the control group (53.3%). A significant difference was observed in the duration of labor, rate of cervical dilatation, augmentation/induction of labor, use of analgesics, and mode of delivery among primigravidae in the experimental and control group at p<0.05. In agreement with these findings, Mirzakhani et al. conducted a study on the effect of birth ball exercises during pregnancy on the mode of delivery in primiparous women and also concluded the same [11]. Similar findings were also reported by Mathew et al. stating a statistically significant difference in the maternal outcome and mode of delivery between the two groups [12].

Limitations of the study

Research findings cannot be generalized to a larger population because of the small sample size. Education and practice for birthing ball exercises during labor were not provided during the antenatal period so mothers found it difficult to follow instructions during labor.

## Conclusions

Though pregnancy and labor are physiological events, addressing maternal discomfort during labor pains is a priority of maternal intervention. The present study has shown that the practice of birthing ball exercise during labor decreased labor pain, and improved favorable labor outcomes among the primigravidae parturient mothers. Therefore birthing ball exercises can prove to be a simple and effective complementary intervention that can be used to manage the discomfort of labor and also to improve its outcome.
